# BKCa participates in E2 inducing endometrial adenocarcinoma by activating MEK/ERK pathway

**DOI:** 10.1186/s12885-018-5027-9

**Published:** 2018-11-16

**Authors:** Fenfen Wang, Qin Chen, Genping Huang, Xuedong Guo, Na Li, Yang Li, Baohua Li

**Affiliations:** 1 Department of Gynecologic Oncology, Women’s Hospital, Zhejiang University, School of Medicine, Xueshi Road 1, Hangzhou Zhejiang, 310006 People’s Republic of China; 2 Department of Pathology, Women’s Hospital, Zhejiang University, School of Medicine, Xueshi Road 1, Hangzhou Zhejiang, 310006 People’s Republic of China

**Keywords:** Endometrial adenocarcinoma, BKCa, MEK1/2, ERK1/2, 17β-estradiol, Prognosis

## Abstract

**Background:**

The large-conductance, voltage-gated, calcium (Ca (2+))-activated potassium channel (BKCa) plays an important role in regulating Ca (2+) signaling and cell physiological function, and is aberrantly expressed in some types of cancers. The present study focuses on identifying the oncogenic potential and clinical significance of BKCa in endometrial adenocarcinoma, as well as exploring the mechanistic relevance by 17β -estradiol (E2) inducing aberrant activation of MEK1/2 and ERK1/2 via BKCa.

**Methods:**

The expression of BKCa, ERK1/2 and p-ERK1/2 were examined by immunohistochemical staining in 263 cases, including 185 primary types I endometrial cancer tissues, 38 atypical endometrial hyperplasia tissues and 40 normal endometrium tissues. Cell growth, cycle, apoptosis rate, migration and invasion was separately tested in Ishikawa cells using siRNA-BKCa and/or E2 treatment, as well as the expression of these interested proteins by western blot analysis.

**Results:**

We showed that expression of BKCa is significantly elevated in 185 types I endometrial adenocarcinoma tissues compared to those of the normal endometrium and atypical endometrial hyperplasia tissues. Furthermore, in vitro observations revealed that down-regulation of BKCa expression inhibited cell growth by both enhancing apoptosis and blocking G1/S transition, suppressed cell migration and invasion in Ishakiwa cells, and decreased the expression of p-MEK1/2 and p-ERK1/2. Additionally, RNAi-mediated knockdown of BKCa attenuated the increased cellular growth and invasion, as well as the elevated expression of p-MEK1/2 and p-ERK1/2 proteins, induced by E2 stimulation. More importantly, the aberrant expression of BKCa and p-ERK1/2 were closely related with poor prognostic factors in type I endometrial cancer, and up-regulated expression of p-ERK1/2 was significantly associated with shorter disease-free survival (DFS) and overall survival (OS) and was an independent prognostic factor in type I endometrial cancer patients.

**Conclusion:**

Our results demonstrated that BKCa and the key downstream effectors p-ERK1/2 could be involved in important signaling pathways in initiation and development of endometrial adenocarcinoma and may provide a new therapeutic approach for women with endometrial cancer.

**Electronic supplementary material:**

The online version of this article (10.1186/s12885-018-5027-9) contains supplementary material, which is available to authorized users.

## Background

Endometrial cancer (EC) is one of the most common malignancies of the female genital tract worldwide. It is grouped into two subtypes; type I, an estrogen-dependent endometrial adenocarcinoma which is associated with a slow progression and good prognosis, whilst type II is characterized as an aggressive non-estrogenic endometrial cancer with a poor prognosis. In general, type I is the most common endometrial cancer subtype [1].

Patients with EC are often diagnosed as early stage, and the best method to balance optimal survival with minimal adverse effects on quality of life has not yet been defined. The 5-year overall survival ranges from 74 to 91% in patients without metastatic disease [1], but the recurrence rate and mortality remains high [2]. Imbalance of estrogen and progesterone leading towards a hyper estrogenic state has been determined to increase the risk of endometrial adenocarcinoma. The mechanisms by which estrogen stimulates the endometrium from atypical endometrial hyperplasia to develop cancer are still unclear. Several studies have demonstrated that abnormal activation of PKCβ /ERK pathway [3], K-Ras/PKCβ [4], PI3K/AKT [5] induced by estrogen were involved in the process. However, the concrete mechanisms by which E2 induces the initiation and progression of endometrial adenocarcinoma have not been fully elucidated. The current study aimed to explore the mechanisms of E2 inducing type I endometrial carcinogenesis and cancerous development, and the related molecular pathway in order to identify a potential new approach for treatment.

The large-conductance, voltage-gated, calcium (Ca (2+))-activated potassium channel (BKCa) is encoded by the KCNMA1 gene and characterized by a large conductance to potassium, plays an important role in regulating Ca (2+) signaling and associates with cell membrane potential and cell metabolism [6, 7]. The altered expressions or activation of BKCa may lead to changes of intracellular calcium and membrane potential resulting in dysfunction of cells and organs. BKCa was revealed as an important molecule in regulating contraction/relaxation of muscle cells [8], neurotransmission in the brain, and metabolism, proliferation, migration, and gene expression [9]. Although it has been described in some types of cancer cell, such as osteosarcoma [10], breast cancer [11] and gastric cancer [12], little is known about the functions of BKCa in uterus. BKCa was studied in uterus smooth muscle and was reported that BKCa is over expressed in adenomyosis tissues, and it might cause abnormal muscle contractility that induces the inflammation effects, endometrium impairment and pain [13]. Moreover, the expression of BKCa in pregnant myometrium is reduced during labor and related with onset of parturition [14, 15]. Recently, Li et al. [16] reported the role of BKCa in endometrial cancer HEC-1-B cells. They demonstrated that forced expression of BKCa could induce proliferation and migration, and the specific opener (NS1619) or blocker (IBTX) of BKCa could regulate the cellular biological behavior. This shows that BKCa could confer growth and migration advantage to endometrial cancer cells. However, there are still no other reports or further investigations about the roles and mechanisms of BKCa in endometrial cancer, especially in the most common subtype endometrial adenocarcinoma.

In the present study, we confirmed elevated expression of BKCa in type I endometrial cancer tissues compared to those in normal endometrium and atypical endometrial hyperplasia tissues, and demonstrated that down-regulated expression of BKCa influenced the cell growth and invasiveness via inactivation of the MEK/ERK pathway. We propose that BKCa, is a membrane protein that not only controls the physiological effects of the endometrial cells, but also participates in the initiation and progression of endometrial adenocarcinoma by E2 stimulation.

## Methods

### Cell culture and siRNA preparation

Human endometrial adenocarcinoma cell line Ishikawa with functional Estrogen Receptor (ER) was purchased from Procell Life Science & Technology Co.,Ltd. (Wuhan, China) (Catalogue numbers: CL-0283) and was further transfected using 50 nM siRNA-BKCa or scramble siRNA. Additional file 1: Table S1 shows sequences of siRNA.

### E2 treatment

17β-estradiol (E2) were purchased from Sigma Company, and dissolved in phosphate buffer solution (PBS) at 1 nM concentration. Then, Ishikawa cells were treated with 1 nM E2 for further study.

### RNA extraction and PCR

Total RNA of cultured cells was isolated by using Trizol reagent. Reverse transcription was performed with the procedure as described previously [17]. PCR reactions for gene expression were performed using SYBR green RT-PCR kit. The primer pairs of BKCa are listed in Additional file 1: Table S1.

### Western blotting analysis

Ishikawa cell lysates were boiled with gel-loading buffer for 5 min at 100 °C, resolved on 10% SDS-PAGE, transferred to PVDF membranes, probed with appropriate antibodies and visualized with enhanced chemiluminescence (ECL). The detailed antibodies information is listed in Additional file 2: Table S2.

### CCK-8 assay

Ishikawa cells were plated in the 96 well plates at a density of 5000 cells per well. At the end of each indicated time point (0, 24, 48, and 72 h post-transfection), cells were incubated with CCK-8 (5 mg/mL) for 4 h at 37 °C and the absorbance read at 570 nm using a micro-plate reader. Each treatment was performed triplicates and each experiment was repeated twice.

### Migration and invasion assay

Migration and invasion assays were carried out as described previously. For migration assays, 5 × 10^4^ cells suspended in 200 μl serum free medium were plated in the upper chamber and incubated at 37 °C for 12 h, while for the invasion assay, 9 × 10^4^cells suspended in 300 μl serum free medium were plated in the upper chamber and incubated at 37 °C for 24 h. Non-invading or migrating cells were scrubbed with a cotton-tip swab and the cells that penetrated through the filter were stained with crystal violet and counted at a magnification of × 200 in randomly selected fields by using a phase-contrast microscope, and the mean number of cells per field was calculated.

### Flow cytometry for cell cycle and apoptosis analysis

Flow cytometry was used to determine cell cycling progression and apoptosis analysis with procedure as described previously. For cell cycling analysis, at 48 h post-transfection, the cells (3 × 10^5^ cells/well) were collected by trypsinization and fixed with ice-cold ethanol (75%) at − 20 °C for 24 h. Then, fixed cells were incubated in a propidium iodide/PBS staining buffer (20 μg/ml propidium iodide,100 μg/ml RNase A, and 0.1% Triton X-100) at 37 °C for 30 min. Data were acquired using Cell Quest software, and the percentages of G0/G1, S, and G2/M phase cells were calculated. For cell apoptosis assays, at 48 h post-transfection of cells (3 × 10^5^ cells/well), Annexin-V and propidium iodide was added to the cells and samples were analyzed within 30 min after staining. Quantification of fluorescence was analyzed by flow cytometry.

### Patients and immunohistochemical staining

The retrospective study enrolled 263 patients, including 185 primary type I endometrial cancer and 38 atypical endometrial hyperplasia tissues, with complete medical record information at Women’s Hospital of Zhejiang University, from January 2007 to December 2009. 40 patients with normal endometrial tissues that underwent the total hysterectomy because of gynecological benign diseases served as controls. The methods of tissue specimen preparation and immunohistochemistry staining performing were described previously [18]. BKCa, ERK1/2 and p-ERK1/2 immunoreactivities were assessed by staining intensity and positively stained distribution of the tumor cells. In brief, staining intensity was scored as negative (0), weakly positive (1), moderately positive (2) or strongly positive (3). The positively stained distribution was scored as 0 for < 0–5%, 1 for 6–25%, 2 for 26–50%, 3 for 51–75%, and 4 for > 75% positively stained cells. Finally, the intensity score and distribution score were multiplied to yield an overall score. A total score < 4 was considered as low expression and ≥ 4 as high expression.

### Follow-up

The patients with type I endometrial cancer were followed up postoperatively by interview at the clinic or by phone call. Regional tumor recurrence, distant metastasis, and patient survival were recorded. Disease-free survival (DFS) and overall survival (OS) were calculated from the day of the surgery until disease recurrence or death. The last day of follow-up was December 2016. The mean follow-up was 94.7 months (range 14–120 months). There were 33 recurrences (33/185, 17.8%) and 30 deaths (30/185, 16.2%).

### Statistical analysis

Statistics were assessed using software packages SPSS version 22.0. For in vitro experiments, a student’s t-test was used to analyze the differences between two groups. The results were presented as Mean ± SD. Correlations between protein expression and the clinic-pathological parameters and significant differences in protein expression were determined by Pearson’s chi-square test. Survival curves were generated by the Kaplan–Meier method and survival rates were compared using the log-rank test. On the univariate and multivariate analyses, the Cox proportional hazard method was used to identify independent predictors of survival. All statistical tests were two-sided, and *p*-values 0.05 were considered statistically significant (* < 0.05, ** < 0.01).

## Results

### Knockdown of BKCa expression influenced the biological effects of Ishikawa cells

**Fig. 1:**
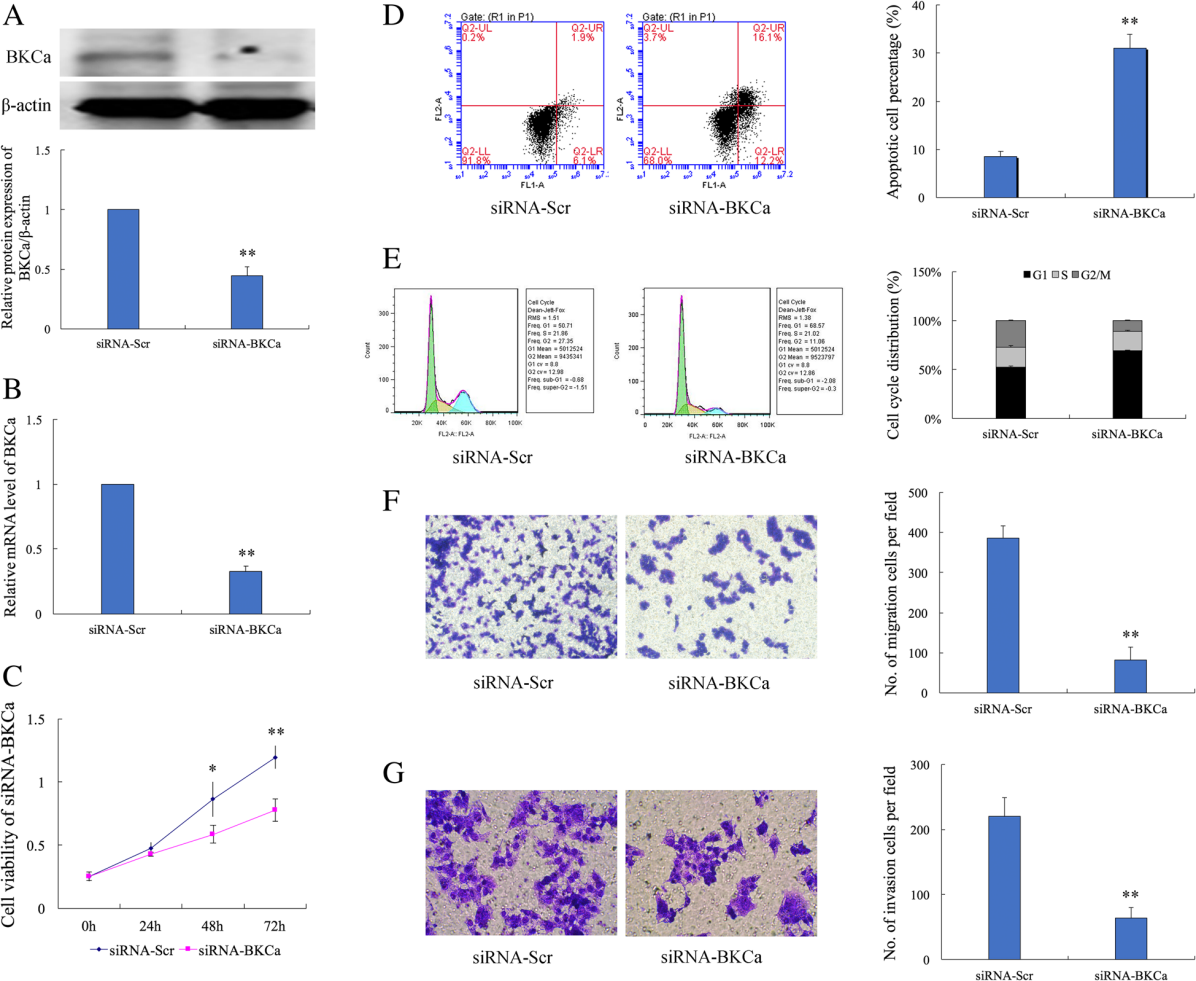
SiRNA targeting BKCa successfully suppressed the expression of BKCa and inhibited the influence of the biological effects of endometrial adenocarcinoma cell line Ishikawa. **a**, **b** The siRNA targeting BKCa effectively down-regulated the mRNA and protein level of BKCa expression at time point of 48 h post-transfection. **c** Knockdown of BKCa significantly inhibited cell proliferation at 48 h and 72 h after transfection of siRNA (*p =* 0.035, *p =* 0.005). **d** At 48 h after transfection, apoptosis assay was performed to determine the apoptotic rate of the two groups of Ishikawa cells. Knockdown of BKCa increased the apoptosis rate from 8.57 ± 1.07% to 30.97 ± 2.93% (*p* = 2.4E^− 4^). **e** FACS was used to analyze the alteration of cell cycle distribution induced by knockdown of BKCa expression. SiRNA targeting BKCa induced blocking of cell cycle progression and resulted in accumulation of G1 stage (*p* = 2.8E^− 4^). **f**, **g** Representative images of migration and invaded cells following knockdown of BKCa expression in Ishikiwa cells were showed by crystal violet staining. SiRNA targeting BKCa reduced significantly the numbers of migration (*p* = 3.18E^− 4^) and invaded (*p* = 0.001) cells. Data (Mean ± SD, *n* = 3 independent experiments)

To investigate the role of BKCa expression in initiation and progression of endometrial adenocarcinoma, we explored the effects of altered expression of BKCa on biological functions of Ishikawa cells. First, we knocked down the expression of BKCa using siRNA, and compared with the negative control. The siRNA targeting BKCa effectively down-regulated the mRNA and protein levels of BKCa at 48 h post-transfection (Fig. 1a, b). We also examined the effects on cellular proliferation using CCK-8 assay, which revealed that inhibition of BKCa expression reduced the proliferation of Ishikawa cells at 48 h and 72 h post-transfection (*p =* 0.035, *p =* 0.005) (Fig. 1c). Secondly, we observed that cell apoptosis was induced by inhibition of BKCa expression in Ishikawa cells as assessed by an Annexin V & PI apoptosis detection kit. The inhibition of BKCa led to increased apoptotic response, and the apoptotic cell rate was significantly elevated compared to the control group (*p* = 2.4E^− 4^) (Fig. 1d). In consistent with apoptosis results, we simultaneously performed the cell cycle pattern by using PI staining and found that down-regulated expression of BKCa triggered a significant accumulation of cells in G1 phase and reduced the percentage of cells in S and G2/M phase (Fig. 1e). This result suggested that BKCa could promote cell growth through influencing cell cycle progression and apoptosis rate. Finally, we determined the effect of BKCa on migratory and invasive capacity in Ishikawa cells using transwell assay. Down-regulated expression of BKCa inhibited the cell migration (*p* = 3.18E^− 4^) and impaired the invasive capacity (*p* = 0.001) (Fig. 1f, g). These observations further support an important role of BKCa in growth and invasiveness of endometrial adenocarcinoma cells and could act as an oncogenic factor contributing to initiation and progression of endometrial adenocarcinoma.

### E2 induced the increase of BKCa expression in Ishikawa cells and promoted cell growth and invasion

Estrogen-ER signaling pathways were found to be crucial for endometrial adenocarcinoma development, and as a steroid hormone E2 was strongly related with the hormone dependent type I endometrial cancer. After stimulating with 1 nM E2, the proliferation of Ishikawa cells was increased significantly at 48 and 72 h (*p =* 0.019, *p =* 0.011) (Additional file 3: Figure S1B), and the rate of apoptosis was decreased when compared to the negative control (*p =* 0.006) (Additional file 3: Figure S1C). Additionally, E2 promoted cell cycle progression through G1 phase (Additional file 3: Figure S1D), and also enhanced the migration (*p =* 5.2E^− 5^) and invasive capacity (*p =* 4.87E^− 4^) of Ishikawa cells (Additional file 3: Figure S1E, F). The MEK/ERK pathways are important molecules in the process of E2 promoting endometrial adenocarcinoma, and the phosphated modifications of MEK1/2 and ERK1/2 are the main active forms for signal transduction. After exposure to 1 nM E2, the total proteins were extracted from the Ishikawa cells, and the interested molecules were examined using western blot. We found that expression of BKCa protein (*p =* 0.013) was significantly up-regulated when compared to the control group. Importantly, the total MEK1/2 and ERK1/2 protein levels were unchanged, whereas the expression of p-MEK1/2 (*p =* 0.049) and p-ERK1/2 (*p =* 0.044) were elevated significantly in Ishikawa cells exposed to 1 nM E2 (Additional file 3: Figure S1A). It suggested that E2 could phosphorylate and active MEK/ERK pathway, leading to effects on a variety of physiologic and development processes in endometrial adenocarcinoma cells. More importantly, the molecular mechanism might be partly dependent regulation of BKCa.

### Knockdown of BKCa attenuated up-regulation of MEK/ERK phosphylation and promotion of Ishikawa growth and invasiveness induced by E2 stimulation

**Fig. 2:**
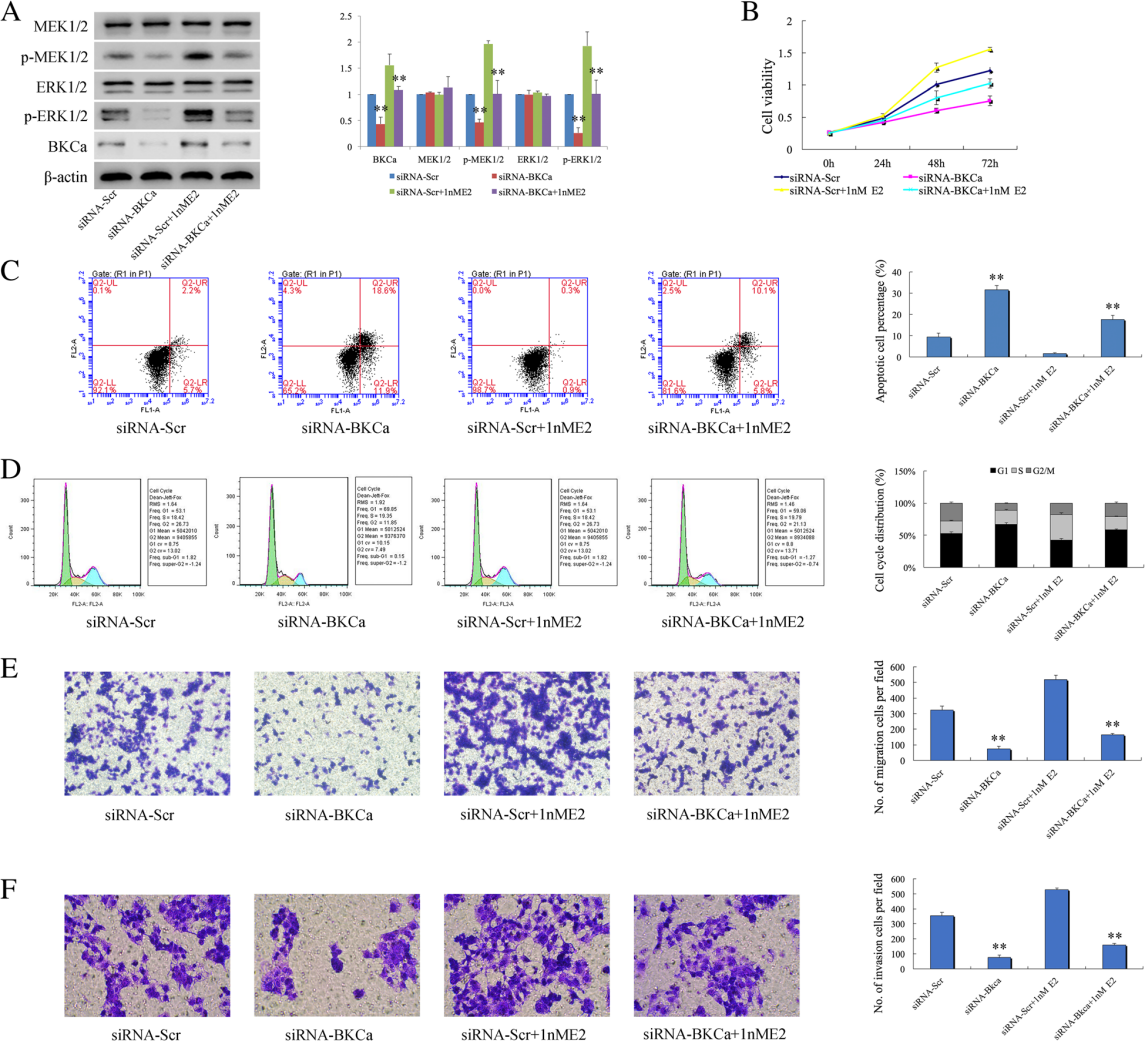
Down-regulation of BKCa expression partially attenuated increased phosphorylation of MEK1/2 and ERK1/2 induced by E2, and reversed the alterations to E2 mediated biological effects in Ishikawa. **a** Down-regulated expression of BKCa using siRNA partially attenuated the increase of p-MEK1/2 (*p* < 0.01) and p-ERK1/2 (*p* < 0.01) expression induced by E2 stimulation. **b** Down-regulated expression of BKCa inhibited the proliferation of Ishikawa cell, whereas 1 nM E2 treatment accelerated the cells growth. Decreased expression of BKca partially attenuated the increased proliferative effects induced by E2 (*p* < 0.05). **c** The reduction in apoptosis by E2 was reversed by siRNA targeting BKCa (*p* < 0.01). **d** Down-regulation of BKCa attenuated the altered cell cycle distribution induced by E2 (*p* < 0.01). **e**, **f** The increased migration and invasion of Ishikawa stimulated by E2 were both reversed by knock down of BKCa expression (*p* < 0.01). Data (Mean ± SD, *n* = 3 independent experiments)

The possibility that BKCa is involved in the process of E2 activating MEK/ERK pathway is still unclear. To investigate the role of BKCa in the molecular mechanism induced by E2, we firstly knocked down the expression of BKCa in Ishikawa cells using siRNA technique, the mRNA and protein expression levels of BKCa were inhibited significantly, meanwhile decreased BKCa expression resulted in the reduction of phosphorylation of MEK1/2 (*p =* 2.96E^− 4^) and ERK1/2 (*p =* 0.001), no change of total MEK1/2 and ERK1/2 protein (Additional file 4: Figure S2A, B). The results showed that BKCa could regulate and activate MEK/ERK pathway in Ishikawa cells. Furthermore, we found that when E2 was used to stimulate the cell growth, the siRNA scramble control group demonstrated increased phosphorylation levels of MEK1/2 and ERK1/2 compared to that without E2 stimulation. However, down-regulation of BKCa with siRNA attenuated increased phosphorylation of MEK1/2 (*p* < 0.01) and ERK1/2 (*p* < 0.01) induced by E2 (Fig. 2a). This suggested BKCa may be an important middle membrane molecule in the process of E2 activating MEK/ERK pathway. Here, we have shown that BKCa plays an important role in growth and invasiveness of Ishikawa cells. When knockdown of BKCa expression using siRNA, that resulted in increased proliferation (Fig. 2b), migration and invasion in Ishikawa cells stimulated by E2 was all reversed accordingly (Fig. 2e, f) (*p* < 0.05). Moreover, the decreased apoptosis and the altered cell cycle distribution have been attenuated(Fig. 2c, d)(*p* < 0.01). This suggests that BKCa could be an essential middle molecular involved in E2 stimulating physiologic and development processes in Ishikawa cells via activating MEK/ERK pathway.

### BKCa and p-ERK proteins closely related with clinicopathological characteristics and prognosis in 185 cases with type I endometrial cancer

We have shown that down-regulation of BKCa expression could induce a decrease in MEK1/2 and ERK1/2 phosphorylation in our study, and ERK was key effector in the pathway. Therefore, immunohistochemistry staining was performed to detect the proteins of BKCa, ERK1/2 and p-ERK1/2 in 263 endometrial tissue samples, including 185 type I endometrial cancer, 40 normal endometrial tissues and 38 atypical endometrial hyperplasia tissues. The detailed information of endometrial adenocarcinoma patients was shown in Additional file 5: Table S3.

**Fig. 3:**
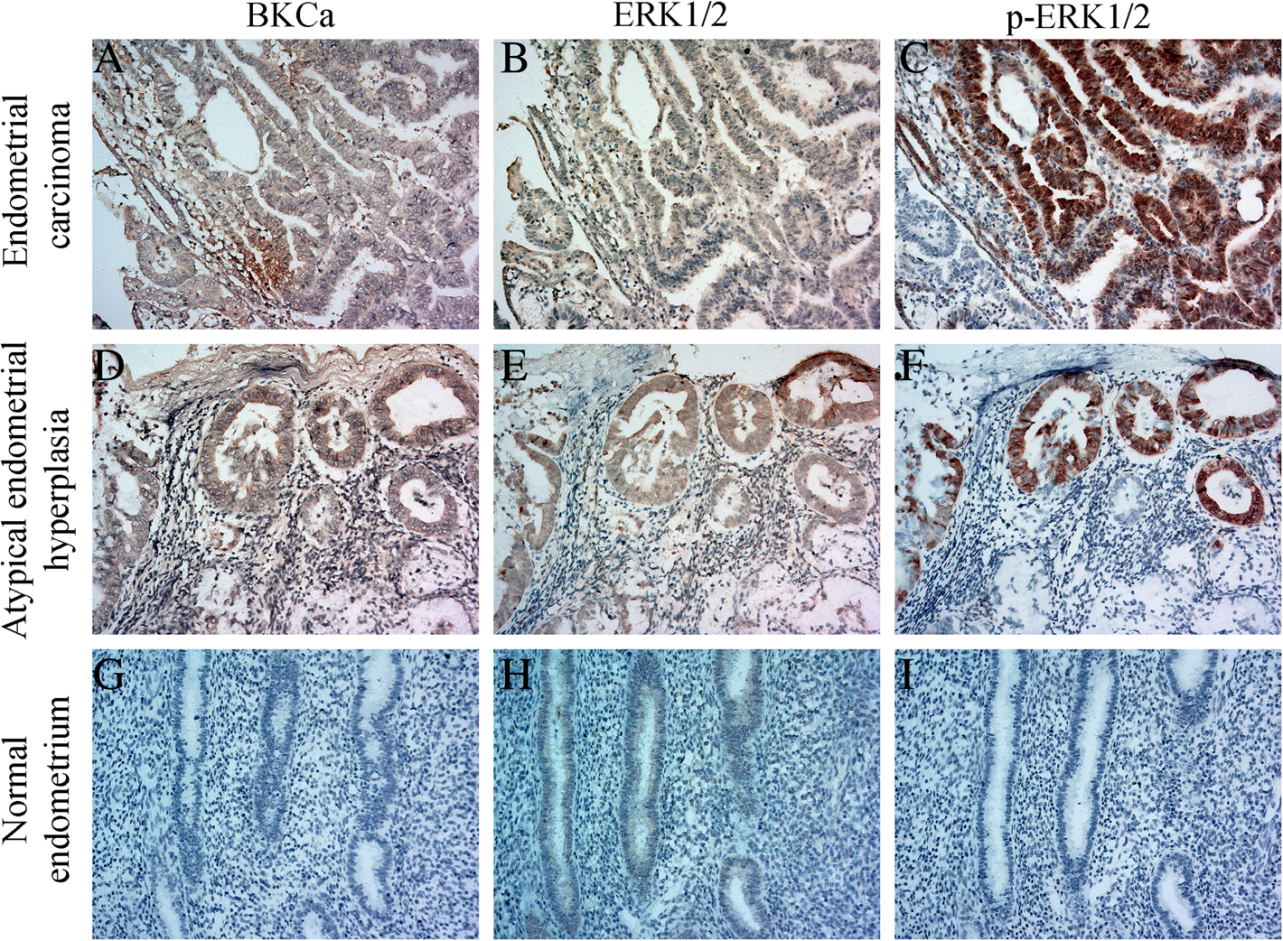
Representative immunohistochemical staining showed BKCa, ERK1/2 and p-ERK1/2 expressions in type I endometrial carcinoma tissues and atypical endometrial hyperplasia tissues using serial section technique. Simultaneously increased BKCa, ERK1/2, p-ERK1/2 expressions were observed in the representative cases with type I endometrial cancer (**abc**) and atypical endometrial hyperplasia tissues (**def**). The negative expressions of these proteins were shown in normal endometrium (**ghi**). Magnification × 200

The representative immunohistochemical staining of BKCa, ERK1/2 and p-ERK1/2 expression is shown in Fig. 3. The majority expressions of endometrial adenocarcinoma tissue showed consistent, strong staining of BKCa of cell membranous throughout all layers. BKCa expression was significantly elevated in endometrial adenocarcinoma tissues compared to those of normal endometrium (*p =* 8.4E^− 5^) and atypical endometrial hyperplasia (*p =* 1.39E^− 4^) (Table.1). There was no significant different in the expression levels of BKCa between the atypical endometrial hyperplasia tissue group and the normal endometrium tissues group (*p* > 0.05). As the key effector molecules on MEK/ERK pathway, the staining of ERK1/2 and p-ERK1/2 was predominantly located in the cytoplasm and nuclear of tumor cells, and they were both over-expressed in endometrial adenocarcinoma compared to normal endometrium (*p =* 4.19E^− 10^, *p =* 2.77E^− 8^) and atypical endometrial hyperplasia tissues (*p =* 1.97E^− 7^, *p =* 0.036) (Table.1). Between atypical endometrial hyperplasia group and normal endometrial group, only the expression of p-ERK1/2 was significantly increased in atypical endometrial hyperplasia (*p* = 0.001), and the expression of ERK1/2 showed no significant differences (Table.1). Further investigations about the correlations of the proteins and the clinico-pathological parameters were analyzed in endometrial adenocarcinoma group. The Pearson chi-square test showed that expressions of ERK1/2 and p-ERK1/2 were positively correlated with BKCa expression (*p* = 0.001, *p* = 0.038) (Table.2). The correlations between the three interested molecules and clinic-pathological parameters in 185 endometrial adenocarcinoma tissues were evaluated by Pearson’s chi-square test. The results from Table.3 showed that the elevated BKCa expression was significantly associated with FIGO stage ≥II (*p* = 0.004) and lymph node metastasis (LNM) (*p* = 0.011). Although ERK1/2 expression was not associated with any clinic-pathological characteristics, the high expression of p-ERK1/2 was significantly related to FIGO stage ≥II (*p* = 0.012), cervical stromal involvement (*p* = 0.014), LVSI (*p* = 0.046) and LNM (*p* = 0.018) (Table.3). It suggested that aberrant expression of BKCa and p-ERK1/2 was closely related with poor prognostic factors in type I endometrial cancer samples.

**Table 1:** The aberrant expressions of BKCa, ERK1/2 and p-ERK1/2 in tissue samples of type I endometrial carcinoma, atypical endometrial hyperplasia and normal endometrium

Proteins	Expression level	Endometrial adenocarcinoma (*n* = 185)	Atypical endometrial hyperplasia (*n* = 38)	Normal endometrium (*n* = 40)
BKCa	High	68	2	2
	Low	117	36	38
ERK1/2	High	110	5	2
	Low	75	33	38
p-ERK1/2	High	93	12	1
	Low	92	26	39

**Table 2:** The correlations of the expressions of ERK1/2, p-ERK1/2 and BKCa proteins in tissue samples of type I endometrial carcinoma

	ERK1/2 expression, n(%)		*p*-value	p-ERK1/2 expression, n(%)		*p*-value
	Low	High		Low	High	
BKCa, n(%)			0.001			0.038
Low	58 (31.4)	59 (31.9)		65 (35.1)	52 (28.1)	
High	17 (9.2)	51 (27.6)		27 (14.6)	41 (22.2)	
ERK1/2, n(%)						0.267
Low				41 (22.2)	34 (18.4)	
High				51 (27.6)	59 (31.9)

**Table 3:** The correlations between the expressions of BKCa, ERK1/2 and p-ERK1/2 proteins and the clinicopathological parameters in 185 cases with type I endometrial cancer

Characteristics	BKCa, n (%)		*p-*value	ERK1/2, n (%)		*p-*value	p-ERK1/2, n (%)		*p-*value
	Low	High		Low	High		Low	High	
Age			0.119			0.151			0.179
≤ 60	82(44.3)	40(21.6)		54(29.2)	68(36.8)		65(35.1)	57(30.8)	
> 60	35(18.9)	28(15.1)		21(11.4)	42(22.7)		27(14.6)	36(19.5)	
FIGO stage			0.004			0.271			0.012
<II	80(43.2)	32(17.3)		49(26.5)	63(34.1)		64(34.6)	48(25.9)	
≥II	37(20.0)	36(19.5)		26(14.1)	47(25.4)		28(15.1)	45(24.3)	
Differentiation			0.733			0.345			0.623
Well/moderate	92(49.7)	52(28.1)		61(33.0)	83(44.9)		73(39.5)	71(38.4)	
Poor	25(13.5)	16(8.6)		14(7.6)	27(14.6)		19(10.3)	22(11.9)	
Myometrial invasion			0.958			0.066			0.201
< 1/2	95(51.4)	55(29.7)		56(30.3)	94(50.8)		78(42.2)	72(38.9)	
≥1/2	22(11.9)	13(7.0)		19(10.3)	16(8.6)		14(7.6)	21(11.4)	
Cervical stromal involvement			0.107			0.457			0.014
No	84(45.4)	41(22.2)		53(28.6)	72(38.9)		70(37.8)	55(29.7)	
Yes	33(17.8)	27(14.6)		22(11.9)	38(20.5)		22(11.9)	38(20.5)	
LVSI			0.104			0.183			0.046
No	112(60.5)	60(32.4)		72(38.9)	100(54.1)		89(48.1)	83(44.9)	
Yes	5(2.7)	8(4.3)		3(1.6)	10(5.4)		3(1.6)	10(5.4)	
LNM			0.011			0.407			0.018
No	114(61.6)	59(31.9)		72(38.9)	101(54.6)		90(48.6)	83(44.9)	
Yes	3(1.6)	9(4.9)		3(1.6)	9(4.9)		2(1.1)	10(5.4)	

*FIGO* International Federation of Gynecology and Obstetrics, *LVSI* lymph vascular space invasion, *LNM* lymph node metastasis

**Fig. 4:**
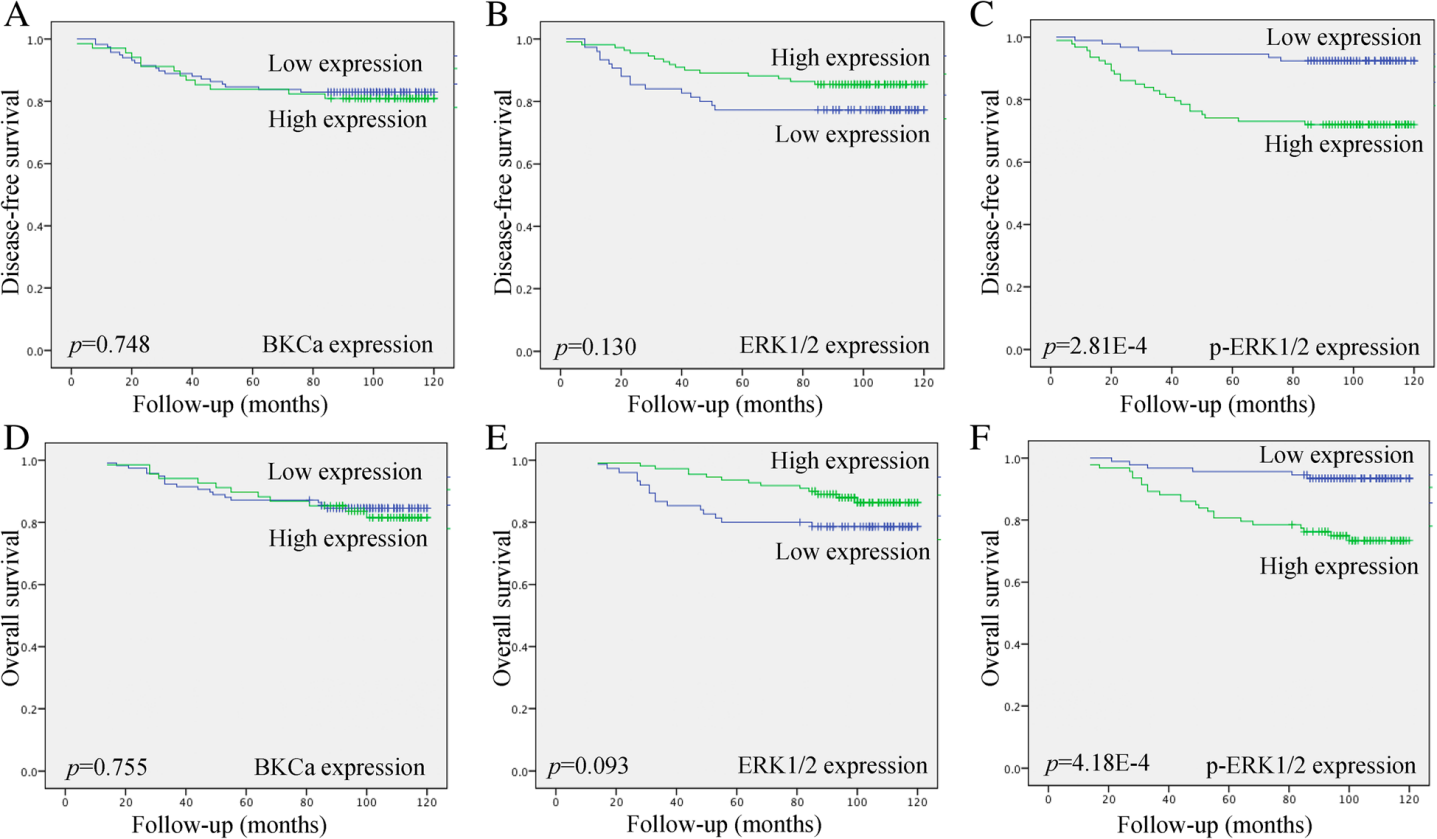
Kaplan–Meier curves showing the association of aberrant expression of BKCa, ERK1/2 and p-ERK1/2 with disease-free survival (DFS) and overall survival (OS) in 185 patients with type I endometrial cancer. High expression of p-ERK1/2 (**c**, **f**) was significantly associated with shorter DFS (*p* = 2.81E^− 4^) and OS (*p* = 4.18E^− 4^) in endometrial adenocarcinoma patients; in contrast, both BKCa and ERK1/2 expressions were not significantly associated with DFS or OS (**a-e**)

Kaplan– Meier analysis was used to investigate the prognostic value of the three proteins. The survival curves showed the association of expression of three proteins with disease-free survival (DFS) and overall survival (OS) in 185 type I endometrial cancer patients. Significance was tested in univariate and multivariate Cox regression models. Interestingly, the expressions of BKCa and ERK1/2 were not significantly associated with DFS or OS. Only up-regulated expression of p-ERK1/2 was significantly associated with shorter DFS (*p* = 2.81E^− 4^) and OS (*p* = 4.18E^− 4^) (Fig. 4). More importantly, univariate and multivariate analysis demonstrated that p-ERK1/2, which could act as downstream effector regulated by BKCa, was an independent prognostic factor of DFS (*p* = 0.004) and OS (*p* = 0.008) in type I endometrial cancer patients (Table.4). This suggests that BKCa and the key downstream effectors p-ERK1/2 could be involved in important signaling pathways in the pathogenesis and development of endometrial adenocarcinoma.

**Table 4:** Univariate and multivariate analysis of the correlations between prognostic value and disease-free and overall survival rates in 185 patients with type I endometrial cancer

Characteristics	Disease-free survival			Overall survival		
	HR	95% CI	*p*	HR	95% CI	*p*
Univariate analysis						
Age	2.603	1.321–5.166	0.006	2.777	1.348–5.718	0.006
FIGO stage	2.956	1.454–6.011	0.003	3.420	1.600–7.312	0.002
Differentiation	5.221	2.628–10.375	2.39E^−6^	5.516	2.676–11.372	3.72E^−6^
Myometrial invasion	4.911	2.477–9.738	5.17E^−6^	5.411	2.635–11.112	4.25E^−6^
LVSI	6.352	2.940–13.722	2.55E^−6^	6.675	3.046–14.627	2.11E^−6^
LNM	14.51	6.994–30.103	6.79E^−13^	15.02	6.964–32.384	4.84E^−12^
Cervical stromal involvement	1.589	0.797–3.170	0.188	1.932	0.942–3.959	0.072
BKCa expression	1.121	0.558–2.253	0.749	1.123	0.541–2.331	0.756
ERK1/2 expression	0.594	0.300–1.175	0.134	0.546	0.267–1.120	0.099
p-ERK1/2 expression	4.149	1.800–9.563	0.001	4.358	1.781–10.664	0.001
Multivariate analysis						
Age	1.717	0.842–3.503	0.137	1.912	0.909–4.025	0.088
FIGO stage	1.268	0.538–2.986	0.587	1.607	0.648–3.983	0.306
Differentiation	3.111	1.413–6.851	0.005	3.494	1.520–8.034	0.003
Myometrial invasion	3.096	1.430–6.703	0.004	3.648	1.642–8.106	0.001
LVSI	0.503	0.154–1.647	0.256	0.635	0.196–2.050	0.447
LNM	5.008	1.513–16.573	0.008	3.832	1.145–12.826	0.029
p-ERK1/2 expression	3.912	1.542–9.927	0.004	3.796	1.415–10.180	0.008

*FIGO* International Federation of Gynecology and Obstetrics, *LVSI* lymph vascular space invasion, *LNM* lymph node metastasis, *HR* hazard ratio, 95% CI, 95% confidence interval

## Discussion

In the present study, we investigated the role of BKCa in the initiation and development of endometrial adenocarcinoma. Our findings indicated that expression of BKCa in endometrial adenocarcinoma tissues were significantly increased compared to those of normal endometrium or atypical endometrial hyperplasia tissues. Additionally, we also found that knock down of BKCa expression in Ishikawa cells inhibited cell proliferation, migration and invasion, and promoted cell apoptosis, as well as blocking cell cycle progression. BKCa has been previously reported to be elevated in many kinds of cancer. BKCa is overexpressed in prostate cancer [19] and up-regulation of BKCa promotes proliferation, migration and invasion of prostate cancer cells. On the contrary, down-regulation of BKCa inhibited growth and metastasis of prostate cancer cells. BKCa was also hypothesized to function as oncoproteins in breast cancer [20], with metastatic breast cancer cells reported to exhibit increased BKCa activity, leading to greater invasiveness and migration [21]. Moreover, BKCa has been reported to act as a potential therapeutic target in breast cancer [22]. BKCa action was mainly mediated through forming a functional complex with alphavbeta3 integrin [19] or coupled with IP3R3 [23] to promote the BKCa activity. There are few studies exploring the relationship between BKCa and endometrial cancer. Recently using HEC-1-B cancer cell line to investigate the role of BKCa in endometrial cancer, Li et al. found that knock down of BKCa significantly inhibited HEC-1-B cell proliferation, migration and tumor growth in vitro and vivo assays [16]. Consistent with the previous reports, our results showed that the expression of BKCa in endometrial adenocarcinoma tissues were increased significantly, with decreased BKCa expression could inhibit cancer cell growth and invasion. It suggested BKCa could act as a potential oncogene in endometrial adenocarcinoma cells. Currently, the alteration and the precise oncogenic mechanisms of BKCa in endometrial adenocarcinoma cells remain unknown.

Accumulating evidence has also confirmed that E2 were important physiological and pathophysiological regulators of uterus and endometrial functions. Moreover, it has been apparent that MEK/ERK pathway is involved in the pathogenesis and development of endometrial cancer acting as the key molecular networkers [24]. E2 could promote the proliferation and stimulate the invasive capability of the endometrial cancer cell lines via the GPR30-mediated MEK/ERK pathway [25]. Autocrine motility factor (AMF) was related with the proliferative and metastatic of endometrial carcinoma and MEK-ERK1/2 pathway was involved [26]. As such, the MEK/ERK pathway may have a potential role in sustaining tumorigenic potential and radio-resistance in Ishikawa cells [27]. However, there are currently few reports about the relationship between estrogen and BKCa in E2 activating MEK/ERK pathway. In the study, we found that E2 increased the expression of BKCa protein, prompted cell growth and invasion, and induced the phosphorylation of MEK1/2 and ERK1/2 proteins. More importantly, the reduction of BKCa expression using siRNA in Ishikawa cells after E2 treatment could significantly attenuate the expression of p-MEK1/2 and p-ERK1/2 proteins, as well as cell growth and invasion capabilities induced by E2 stimulation, consequently suggesting that BKCa at least partially participates in E2 inducing endometrial adenocarcinoma by activating MEK/ERK pathway. A previous report has shown that the G-protein coupled estrogen receptor 1 (GPER1) stimulation, that is activated by estrogen, could result in an increased BKCa channel activity. BKCa is directly activated by estrogens, which have an essential role in cancers of breast [28] and prostate through both genomic and non-genomic mechanisms. Estrogen at physiological concentration enhances BKCa channel activity through CGMP-PKG signaling pathway by stimulating generation of NO in vascular smooth muscle cells (VSMCs) [29]. Chronic exposure to estrogen also alters BKCa channel function in VSMCs. Estrogen could stimulate the transcriptional promoter activity of the same gene in mouse through activation of estrogen receptor α (ERα) [30] by binding to the estrogen responsive sequences in the promoter of mouse α-subunit gene. 17β-estradiol in an ex vivo tissue culture system augmented BKCa channel activity, which was accompanied by increased protein expression of β1 subunit [8], and the similar treatment of ovarectomized guinea pig increased expression of α subunit in aorta [31, 32]. However, on the contrary, exposure of cultured human coronary arterial myocytes to a pharmacological concentration of 17β-estradiol led to downregulation of BKCa channels [33]. Currently, the detailed mechanism about how estrogen mediated increase of BKCa is still not clear. It appears that estrogen augmented expression of BKCa is species specific or tissue dependent. Though we did not reveal the mechanism about E2 inducing elevated expression of BKCa in the current study, it may be similar to the previous studies.

It is well established that the occurrence of endometrial adenocarcinoma is mainly stimulated by estrogen, and progresses slowly. The epithelium transforms from normal tissue through atypical endometrial hyperplasia to develop endometrial cancer. Atypical endometrial hyperplasia can be characterized as a pre-cancerous disease, and the majority of them progress to invasive disease if without effective clinical treatment. However, there is a current lack of biomarkers to predict progression to endometrial cancer. In the study, the data showed that the expressions of BKCa, ERK1/2 and p-ERK1/2 were significantly increased in endometrial adenocarcinoma compared to normal endometrium or atypical endometrial hyperplasia tissues. More importantly, only a gradual increase of p-ERK1/2 expression was observed beginning in normal endometrium and continuing through atypical endometrial hyperplasia to endometrial adenocarcinoma. Hence, Increased p-ERK1/2 expression closely related to clinical characteristics and might act as a potential biomarker to predict the occurrence of endometrial adenocarcinoma.

Though the prognosis of early stage endometrial adenocarcinoma with primary surgical resection is relative good, improvement of the five-year survival rate is still the goal of the clinical members. We have known that several molecular alterations had been described in the different types of EC, and these genes involved in the important signaling pathways were key factors related with the prognosis of the disease. The most relevant altered pathways in EC, include PI3K/AKT/mTOR [24], RAS-RAF-MEK-ERK [34], Tyrosine kinase, WNT/β-Catenin [35], and TGF-β signaling pathways [36]. MEK/ERK pathway is also an important kinase involved in the signal transduction of endometrial carcinoma and act as key targets in many cancer biological target therapies. ERK, as a key effector in MEK/ERK pathway, can be phosphorylated and translocated into the nucleus that inducing activation of downstream transcriptional factors to participate in the regulation of growth, development and differentiation. On the other hand, the level of p-ERK was found to be closely related to the prognosis of gastric cancer, hepatic cancer, and colon cancer. Hence, p-ERK acts as a prognostic factors and key oncogenic gene in different type of malignant cancer, with previous studies reporting that the elevated expression of p-ERK was correlated with later stage, deeper invasion and poor differentiation in endometrial cancer [34]. In the study, we found that expressions of ERK1/2 and p-ERK1/2 were both positive correlated with the expression of BKCa in 185 cases with type I endometrial cancer, and BKCa could regulate the expression of p-MEK1/2 and p-ERK1/2 to influence cell growth and invasion in endometrial cancer cells. The present study is the first time showed possible association of BKCa with MEK/ERK pathway in endometrial adenocarcinoma. In the study, though BKCa was not an independent prognostic factor, BKCa could regulate the expression of p-ERK1/2 that could serve as an independent prognostic predictor of shorter DFS and OS in endometrial adenocarcinoma patients by univariate and multivariate analysis. Taken together, we suggest that BKCa acts as an oncogenic activity membrane molecule and could regulate MEK/ERK pathway to participate in the initiation and development of endometrial adenocarcinoma and influence the prognosis and survival rate.

## Conclusions

In summary, BKCa had been confirmed to be an essential membrane molecule acting as an oncogenic factor in endometrial adenocarcinoma cell and regulate the p-MEK1/2 and p-ERK1/2 proteins expression to participate in cell growth and invasion by E2 stimulation. Therefore, targeting BKCa by siRNA could effectively inhibit the activity of MEK/ERK pathway and attenuate the cellular behavior induced by E2 treatment. These novel findings promote a better understanding of the functions and mechanisms of BKCa in endometrial adenocarcinoma, and may provide a new approach for the treatment of endometrial adenocarcinoma patients.

## Additional files

Additional file 1: **Table S1.** The information about the sequences. (DOCX 16 kb)

Additional file 2: **Table S2.** The information of the primary antibodies used in the studies. (DOCX 15 kb)

Additional file 3: **Figure S1.** 17β-estradiol (E2) induced an increase in the expression of BKCa in Ishikawa cells and promoted the cell growth and invasion. (A) Western Blot showed that 1 nM E2 treatment significantly increased level of BKCa protein (*p =* 0.013) and the phosphorylation of MEK1/2 (*p =* 0.049) and phosphorylation of ERK1/2 (*p =* 0.044) in Ishikawa cells, but had no effect on level of total MEK1/2 and ERK1/2. (B) Exposure to 1 nM E2 promoted cell proliferation rate at 48 h and 72 h in Ishikawa cells (*p =* 0.019, *p =* 0.011). (C) Apoptosis assay was performed to determine the early and late apoptotic rate induced by E2 treatment in Ishikawa cells. 1 nM E2 reduced the apoptosis rate from 3.11 ± 0.53% to 1.30 ± 0.26% (*p =* 0.006). (D) FACS was used to analyze the alteration of cell cycle distribution induced by E2 treatment. 1 nM E2 promoted cell cycle progression and induced increased percentage of cells in S phase and decreased number of cells of G1 and G2/M stage percent. (E, F) 1 nM E2 also increased the numbers of migration (*p =* 5.2E^− 5^) and invaded (*p =* 4.87E^− 4^) cells significantly in Ishikawa cells. Data (Mean ± SD, *n* = 3 independent experiments). (TIF 51687 kb)

Additional file 4: **Figure S2.** Knockdown of BKCa expression using siRNA significantly decreased the phosphorylation level of MEK1/2 (*p =* 2.96E^− 4^) and ERK1/2 (*p =* 0.001) in Ishikawa, but had no effect on level of the total MEK1/2 and ERK1/2 expressions. (TIF 7103 kb)

Additional file 5: **Table S3.** The information about the tissue samples of the patients with type I endometrial cancer and the related clinic-pathological parameters. (DOCX 16 kb)

## Abbreviations

BKCa: The large-conductance, voltage-gated, calcium (Ca (2+))-activated potassium channel
DFS: Disease-free survival
E2: 17β–estradiol
EC: Endometrial cancer
ER: Estrogen Receptor
FIGO: The International Federation of Gynecology and Obstetrics
LNM: Lymph node metastasis
LVSI: Lymph vascular space invasion
OS: Overall survival
