# Supplementary material for: BKCa participates in E2 inducing endometrial adenocarcinoma by activating MEK/ERK pathway
Source: BMC Cancer. 2018 Nov 16;18:1128. doi: 10.1186/s12885-018-5027-9 (PMC6240221; doi:10.1186/s12885-018-5027-9)
Supplement: Supplementary file 1 — Table S1. The information about the sequences. (DOCX 16 kb) [file 12885_2018_5027_MOESM1_ESM.docx]

Table S1. The information about the sequences

|  | Gene name |  | Sequence (5’-3’) |
| --- | --- | --- | --- |
| siRNA sequences | siRNA-BKCa | sense sequence | GCUCAAGCUCCUAAUGAUAdTdT |
|  |  | antisense sequence | UAUCAUUAGGAGCUUGAGCdTdT |
| RT-PCR primers | BKCa gene | forward primer | GAATGGGAGACGCTTCATAAC |
|  |  | reverse primer | CTGATTGGCTGACAGGATAAC |
|  | β-actin gene | forward primer | CATCGTCCACCGCAAATGCTTC |
|  |  | reverse primer | AACCGACTGCTGTCACCTTCAC |
