# Supplementary material for: BKCa participates in E2 inducing endometrial adenocarcinoma by activating MEK/ERK pathway
Source: BMC Cancer. 2018 Nov 16;18:1128. doi: 10.1186/s12885-018-5027-9 (PMC6240221; doi:10.1186/s12885-018-5027-9)
Supplement: Supplementary file 2 — Table S2. The information of the primary antibodies used in the studies. (DOCX 15 kb) [file 12885_2018_5027_MOESM2_ESM.docx]

Table S2. The information of the primary antibodies used in the studies

| Protein names | Information |
| --- | --- |
| BKCa | Abcam (Ab99046) |
| MEK1/2 | Affinity (AF6384) |
| p-MEK1/2 | Affinity (AF3384) |
| ERK1/2 | Abgent (AM2189b) |
| p-ERK1/2 | Abgent (AP3906a) |
| β-actin | CST (#4970) |
| Secondary antibodies | Biotime company (A0208, A0181, A0216) |
