# Supplementary figures and images for: BKCa participates in E2 inducing endometrial adenocarcinoma by activating MEK/ERK pathway
Source: BMC Cancer. 2018 Nov 16;18:1128. doi: 10.1186/s12885-018-5027-9 (PMC6240221; doi:10.1186/s12885-018-5027-9)

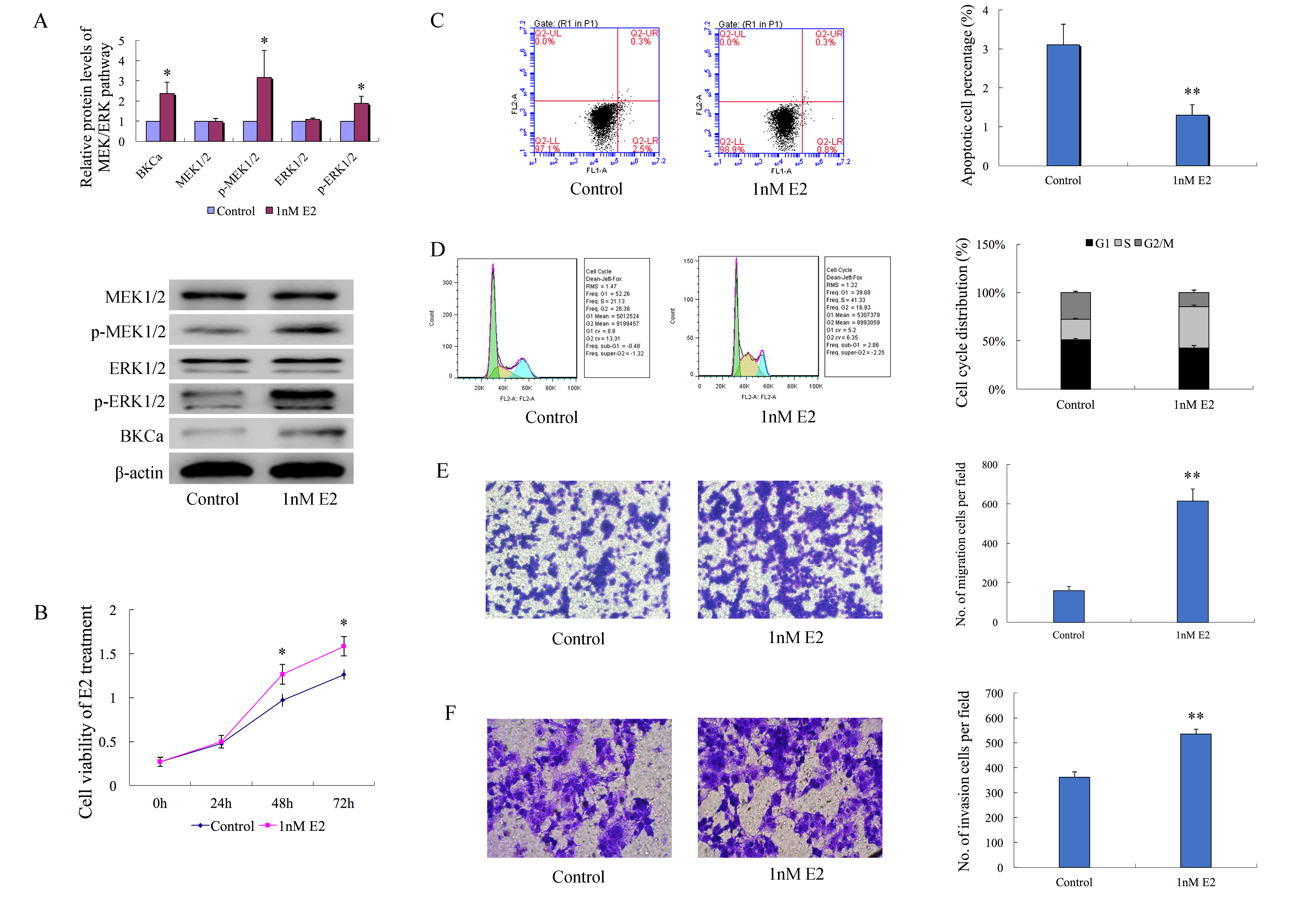

Supplement: Supplementary file 3 — Figure S1. 17β-estradiol (E2) induced an increase in the expression of BKCa in Ishikawa cells and promoted the cell growth and invasion. (A) Western Blot showed that 1 nM E2 treatment significantly increased level of BKCa protein (p = 0.013) and the phosphorylation of MEK1/2 (p = 0.049) and phosphorylation of ERK1/2 (p = 0.044) in Ishikawa cells, but had no effect on level of total MEK1/2 and ERK1/2. (B) Exposure to 1 nM E2 promoted cell proliferation rate at 48 h and 72 h in Ishikawa cells (p = 0.019, p = 0.011). (C) Apoptosis assay was performed to determine the early and late apoptotic rate induced by E2 treatment in Ishikawa cells. 1 nM E2 reduced the apoptosis rate from 3.11 ± 0.53% to 1.30 ± 0.26% (p = 0.006). (D) FACS was used to analyze the alteration of cell cycle distribution induced by E2 treatment. 1 nM E2 promoted cell cycle progression and induced increased percentage of cells in S phase and decreased number of cells of G1 and G2/M stage percent. (E, F) 1 nM E2 also increased the numbers of migration (p = 5.2E− 5) and invaded (p = 4.87E− 4) cells significantly in Ishikawa cells. Data (Mean ± SD, n = 3 independent experiments). (TIF 51687 kb) [file 12885_2018_5027_MOESM3_ESM.tif]

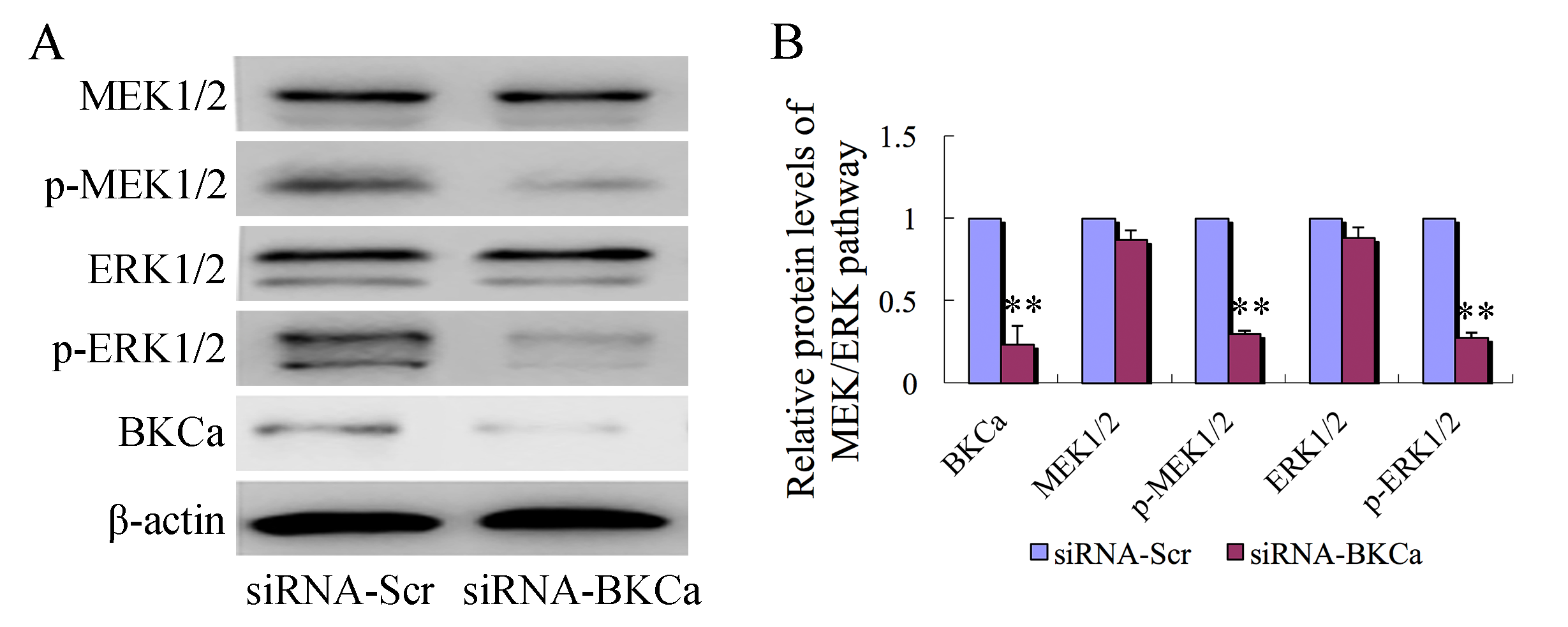

Supplement: Supplementary file 4 — Figure S2. Knockdown of BKCa expression using siRNA significantly decreased the phosphorylation level of MEK1/2 (p = 2.96E− 4) and ERK1/2 (p = 0.001) in Ishikawa, but had no effect on level of the total MEK1/2 and ERK1/2 expressions. (TIF 7103 kb) [file 12885_2018_5027_MOESM4_ESM.tif]
