# Supplementary material for: BKCa participates in E2 inducing endometrial adenocarcinoma by activating MEK/ERK pathway
Source: BMC Cancer. 2018 Nov 16;18:1128. doi: 10.1186/s12885-018-5027-9 (PMC6240221; doi:10.1186/s12885-018-5027-9)
Supplement: Supplementary file 5 — Table S3. The information about the tissue samples of the patients with type I endometrial cancer and the related clinic-pathological parameters. (DOCX 16 kb) [file 12885_2018_5027_MOESM5_ESM.docx]

Table S3 The information about the tissue samples of the patients with type I endometrial cancer and the related clinic-pathological parameters

| Pathological parameters | classification | n=185 (%) |
| --- | --- | --- |
| Age | Age ≤ 60 | 122 (65.9%) |
|  | Age > 60 | 63 (34.1%) |
| FIGO stage | I | 112 (60.5%) |
|  | II | 51 (27.6%) |
|  | III | 20 (10.8%) |
|  | IV | 2 (1.1%) |
| Differentiation | well | 19 (10.3) |
|  | moderate | 125 (67.6%) |
|  | poor | 41 (22.2%) |
| LNM | Yes | 12 (6.5%) |
|  | No | 173 (93.5%) |
| LVSI | Yes | 13 (7.0%) |
|  | No | 172 (93.0%) |
| Myometrial invasion | <1/2 | 150 (81.1%) |
|  | ≥1/2 | 35 (18.9%) |
| Cervical stromal involvement | Yes | 60 (32.4%) |
|  | No | 125 (67.6%) |

FIGO, International Federation of Gynecology and Obstetrics; LVSI, lymph vascular space invasion; LNM, lymph node metastasis.
